# Multifunctional Magnetic Nanocomposites: Innovative Processing and Applications

**DOI:** 10.3390/nano13010206

**Published:** 2023-01-03

**Authors:** Victor Kuncser

**Affiliations:** National Institute of Materials Physics, Atomistilor 405A, 077125 Magurele, Romania; kuncser@infim.ro

## Abstract

Multifunctional magnetic nanocomposites are among those heterogeneous nanosized systems where at least one phase component is magnetic and can act as an intermediate of either the actuation or the response of the overall system. The main advantage of heterogeneous nanosystems is the possibility of combining and inter-influencing the electronic properties of constituent interfaced nanophases. Consequently, unique physico-chemical properties of the hybrid materials of interest in various applications can be obtained. This Special Issue of *Nanomaterials* highlights the most advanced processing and characterization tools of some multifunctional magnetic nanocomposites and heterogeneous systems of interest in various applications, from biomedicine to sensoristics and energy-saving materials.

The first class of heterogeneous systems consists of functionalized magnetic nanoparticles (MNPs) dispersed in different media for biomedical applications, especially of interest in hyperthermia. The fist paper in this Special Issue, by Liliana P. Ferreira et al. [1], reports the additive assisted synthesis of coated iron oxide MNPs for magnetic hyperthermia. The magnetic nanoparticles were synthesized via co-precipitation without or with different additives, using eco-friendly conditions and materials for a green synthesis process. Optimized water-based suspensions of the MNPs with respect to both stabilization and intrinsic loss power (ILP) performances were prepared by using peptine as an additive. Further on, Joana Goncalves et al. report the synthesis and characterization of magnetite nanoparticles coated with fucoidan [2], which is a biopolymer with recognized biocompatibility and tumoral activity. To assess the hyperthermia efficiency, stable suspensions were prepared at a pH close to that of human blood. It has been shown that the obtained ILP values for suspensions of magnetite nanoparticles (6–12 nm in diameter), coated with fucoidan, are usually higher than the ones reported in the literature for magnetite MNPs of various sizes, non-coated or coated with other polymers. A table summarizing the ILP values for magnetite MNPs non-coated or coated with other polymers, for biomedical applications, is also provided in the mentioned report. Moreover, the heating efficiency of the system of magnetite MNPs coated with fucoidan is developed under the biocompatibility criterion of for the used AC magnetic field (i.e., specified upper limits for its amplitude and frequency).

It is worth mentioning that even when respecting the biocompatibility criterion for the applied AC magnetic field, a certain non-negligible heating can also be induced in the conductive healthy tissues inside the actuating coil, due to Eddy currents. This additional heating induced in the healthy tissues unloaded with MNPs is usually neglected and almost no reports on its reduction or comparison to the active heating by MNPs are mentioned in the literature. This time, Balousis and co-authors carefully considered the improvement of the magnetic hyperthermia treatment with MNPs via reduction of the Eddy currents in healthy tissues [3]. Two protocols were considered in this respect, based on the movement of the electromagnetic coil (heating source) with respect to the tumor-bearing tissue. This one has a much lower volume as compared to the coil volume and is initially placed on the coil symmetry axis. Optimal results were obtained under a symmetrical linear motion of the coil, left and right with respect to the tumor-bearing tissue, perpendicular to the symmetry axis of the coil. According to their estimation, the temperature rise under the upper limits of the applied AC magnetic fields respecting the biocompatibility criterion is reduced by 25% in the healthy tissue and only by 1% in the tumor tissue loaded with MNPs. 

Fe-Rh MNPs of equiatomic composition are of interest in many applications, from heat-assisted magneto-recording to magneto-caloric cooling, catalysis and magnetic hyperthermia. It is a real multifunctional material, with many of its specific properties related to the first order antiferro–ferromagnetic phase transition appearing at a temperature close to 370 K in the body-centered cubic structure. This transition is accompanied by an increase in entropy and net magnetic moment and a decrease in the electrical resistivity. However, in the chemically disordered equiatomic phase of Fe-Rh, a face-centered cubic structure (γ-FeRh) with antiferromagnetic order can be induced and exploited in different applications. Ruksan Nadarajaj et al. reported in [4] the synthesis of FeRh MNPs via pulsed laser ablation in liquids (LAL). Being considered a combined top-down and bottom-up synthesis method to produce colloidal nanoparticles, LAL usually leads to the formation of metal oxide nanoparticles or metal complexes during the ablation of the metallic target. Therefore, the focus in [4] was on controlling the oxidation of magnetic and electrically conductive solid solution of FeRh nanoparticles during the LAL synthesis process. In specific conditions, metallic γ-FeRh nanoparticles with monomodal size distribution, or a mixture of γ-FeRh nanoparticles and body-centered cubic FeRh nanoparticles, which are possible to be collected by magnetophoretic separation, were obtained and subsequently characterized in detail. 

A step forward in the preparation of metallic MNPs, this time in a conductive matrix, was provided in [5]. Claudiu Locovei et al. report on the magnetron sputtering co-deposition of Fe–Au nanophase thin films of different compositions and thicknesses. It has been proven by complex investigations that different configurations of α-Fe MNPs (about 4 nm in size) can be formed in the Au matrix depending on the preparation conditions, in close relation to the formation of the unusual hexagonal phase of Au. The as-obtained magnetic configurations, from randomly distributed MNPs to auto-assembled MNPs in lamellar or filiform configurations, were investigated in detail with respect to different magneto-functionalities and magnetic behaviors [5]. Different types of magnetic order, from the 2D Ising type to the 3D Heisenberg type, were evidenced and explained via the involved types of inter-cluster magnetic interactions and spin anisotropies. 

A new Zn phosphate–tellurite glass for magneto-optical applications was reported in [6] by Mihai Elisa et al. The obtained glass, seen as a complex composite system consisting of a non-crystalline vitreous phase and Te-based nanoclusters surrounded by oxygen vacancies, shows a significant positive Faraday rotation angle which decreases with the wavelength. Hence, the system is proposed as an alternative to the more expensive paramagnetic glasses highly doped by expensive rare-earth magnetic elements. 

Of significant interest for different applications, from visible light water splitting to a new generation of random-access memories, is the multiferroic gallium iron oxide, (GFO). GFO, with the formula GaFeO_3_, presents both ferromagnetic and ferroelectric properties and crystallizes in the orthorhombic structure. Different preparation routes have been reported for the preparation of GFO, involving either solid-state reactions or chemical routes. Lucian Diamandescu et al. report in [7] a multifunctional GFO system obtained via mechanochemical activation followed by calcination of the equimolar nanosystem Ga_2_O_3_-Fe_2_O_3_. The most suitable ball milling conditions to obtain the nanoscale GFO ortho-ferrite were reported, as well as a complex characterization of the intermediate and final compounds. Possible applications as a photocatalytic material were also suggested. 

Alina Crisan et al. reported on the synthesis, morpho-structural characterization, thermal stability and superparamagnetic behavior of Mn–Al–C melt-spun ribbons [8]. It is well known that the Mn–Al binary alloy has recently attracted significant interest as a potential solution for low-cost, rare-earth-free, permanent magnets. The large anisotropic field in intermetallic Mn–Al is related to the formation of the phase-centered tetragonal fct τ-phase, in close similarity with the formation of the fct L1_0_ phase of FePt or CoPt alloys. While different phases can appear in Mn–Al or Fe–Pt alloys, and only one type is effective for hard magnetic properties, it becomes interesting to monitor the phase transformation and phase stability in Mn–Al melt-spun ribbons (whose degrees of freedom are additionally increased by the third element, C) with respect to processing conditions and thermal treatments. Finally, the magnetic behavior in correlation to structural changes and phase composition and stability was reported in [8].

Not only the hard magnetic properties are of interest, as intimately related to the formation of the fct L1_0_ phase in FePt alloys, but also the soft magnetic properties, usually related to the formation of the face-centered cubic structure specific to relatively lower concentrations of Fe. The fabrication and detailed characterization of anomalous Hall sensors based on soft magnetic Fe_x_Pt_1-x_ thin films with large Hall angle were reported by Kang Wang et al. in [9]. Both magneto-transport and noise characterizations were performed. The optimal thickness and composition of the Fe_x_Pt_1-x_ thin films leading to energy-efficient anomalous Hall sensors of interest in micro-sensing applications were obtained.

The coexistence of hard and soft magnetic phases in Fe–Pt nanocomposite magnets leading to efficient interfacial exchange-spring effects was reported by Ovidiu Crisan et al. in [10]. Two Fe_x_Pt_1-x_ alloys, with x = 0.53 and 0.55, were prepared by dynamic rotation switching and ball milling, and subsequent annealing at various temperatures. Maximum energy products comparable with specific values of present performing permanent magnets were obtained on samples annealed at 500 °C, demonstrating a new route to obtain rare-earth-free nanocomposite magnets with interfacial exchange-coupled hard–soft magnetic nanophases. Further on, exchange-coupled nanocomposites consisting of SmCo_5_ + 10 wt% Fe were reported by Arnab Chakraborthy et al. in [11]. It should be noted that the SmCo_5_ precursor was obtained from commercial magnets recycled by hydrogen decrepitation. The energy product in optimized processing conditions approaches 95% of the value obtained when using virgin SmCo_5_, showing a new route for the sustainable production of nanostructures exchange-coupled industrial magnets. 

Finally, the last class of reported heterogeneous nanostructured systems consists of interfaced ferromagnetic and antiferromagnetic nanophases presenting specific magneto-functionalities related to induced unidirectional magnetic anisotropy. Unidirectional magnetic anisotropy in molybdenum dioxide, MoO_2_, and hematite, α-Fe_2_O_3_, mixed oxide nanostructures was reported by Felicia Totea et al. in [12]. The aim of the work was to extend the research on diluted magnetism by a comprehensive study on the possibility to control the room temperature ferromagnetism of MoO_2_ by the interfacial magnetic interactions with the antiferromagnetic phase of hematite in equimolar mixtures of MoO_2_ and α-Fe_2_O_3_ nanoparticles ball-milled for different amounts of time. The observed substantial exchange bias effects of the nanocomposite mixtures were interpreted in terms of the unidirectional anisotropy induced by pinning the long-range interacting net magnetic moments of defect hematite nanoparticles (i.e., without the Morin transition), as mediated by the diluted ferromagnetism of MoO_2_, to those less defect hematite nanoparticles (i.e., supporting the Morin transition). Unidirectional magnetic anisotropy effects were also reported by Claudiu Locovei et al. in dense, vertically standing arrays of passivated Ni nanotubes (Ni NTs) [13]. Ni NTs with different thicknesses of the walls were obtained by electrochemical synthesis. Ultra-thin antiferromagnetic layers of NiO were formed on the internal walls of the Ni NTs by further passivation. The experimental evidence and magnetic implications of the unidirectional anisotropy in Ni NTs with cylindrical ferromagnetic/antiferromagnetic interfaces were provided for the first time. 
